# The Effect of Three Different Strategies Based on Motor Task Performance on Neuromuscular Fatigue in Healthy Men and Men with Multiple Sclerosis

**DOI:** 10.3390/medicina54030033

**Published:** 2018-05-24

**Authors:** Laura Kyguolienė, Albertas Skurvydas, Nerijus Eimantas, Neringa Baranauskienė, Renata Balnytė, Marius Brazaitis

**Affiliations:** 1 Department of Applied Biology and Rehabilitation, Lithuanian Sports University, Kaunas 44221, Lithuania; albertas.skurvydas@lsu.lt (A.S.); marius.brazaitis@lsu.lt (M.B.); 2 Sports Science and Innovation Institute, Lithuanian Sports University, Kaunas 44221, Lithuania; nerijus.eimantas@lsu.lt (N.E.); neringa.baranauskiene@lsu.lt (N.B.); 3 Department of Neurology, Medical Academy, Lithuanian University of Health Science, Kaunas 44307, Lithuania; renatabalnyte@gmail.com

**Keywords:** multiple sclerosis, motor task specificity, motor accuracy, subjective force sensations

## Abstract

*Background and objectives*: Fatigue during physical activity occurs because of decreased neuromuscular function. The aim of this study was to evaluate the effect of three different strategies based on motor task performance on neuromuscular fatigue in healthy men and men with multiple sclerosis (MS). *Materials and Methods*: We studied age-matched (18–43 years of age) healthy men (*n* = 15) and men with MS (*n* = 9). The inclusion criteria for MS subjects were a Kurtzke Expanded Disability Status Score <4 and a Fatigue Severity Scale Score >5. Both groups performed one of three exercise trials (with at least a 1-week interval between them) of 100 intermittent isometric knee extensions with flexion of 60°. The three different experimental conditions (ECs) were intermittent isometric contraction tasks with constant, predictable, and unpredictable torque target sequences. The variation of maximal voluntary contraction contractions (MVCs) within the strategies was 25%, 50%, and 75%, with a set average of 50%. All of them had a 5 s contraction and a 20 s rest period. The variables were measured: before exercise, after 100 repetitions (100-Reps), and 1 h after exercise. *Results*: In all EC tasks, the central activation ratio values of healthy and MS subjects were significantly different; however, no significant differences were observed among the EC tasks. No significant differences were seen in electrically induced torque, MVC torque, muscle temperature, subjective sensation of effort, coefficient of variation, or constant and absolute error after 100-Reps and 1 h after exercise between the two groups and in all EC tasks. *Conclusions*: Men with MS experienced higher central motor fatigue than did healthy men, but this had no effect on the variability, accuracy, or force sensation of the movements performed.

## 1. Introduction

Fatigue occurs because of exercise-induced limitations in skeletal muscles or in the nervous system [1,2,3,4,5]. The terms “central fatigue” and “peripheral fatigue” have been used to differentiate these two possible places of muscle fatigue [1,3]. Fatigue is one of the most common symptoms of multiple sclerosis (MS) and can have a major effect on health-related quality of life [6,7]. However, no universal agreement has been reached concerning the formal definition of fatigue, and its causes and mechanisms are not understood.

It has been reported that persons with MS experience increased motor fatigue compared with healthy control persons during constant, repetitive contractions and ambulation [3,8,9]. Strong evidence suggests that the fatigue associated with MS results from reduced voluntary activation of muscles via central mechanisms [3,8,10].

Exercise can be a helpful rehabilitation approach for individuals with MS to reestablish function, endorse wellness, and increase involvement in activities of daily living [11,12,13]. Moreover, accumulating evidence suggests that persons with MS are less physically active than are non-diseased populations [12].

Evidence exists for endorsing involvement in endurance training at low-to-moderate intensity, as the existing literature reveals that persons with MS can both tolerate and benefit from this training modality [14]. In addition, moderate-intensity resistance training also seems to be well tolerated and to have beneficial effects on patients with MS [15,16].

Recently, we found that intermittent isometric contractions (IICs) increased central and peripheral fatigue, force sensation, and intramuscular temperature and diminished absolute and constant error with no visual feedback (VF), but had no effect on motor variability [17] in healthy persons. Insignificant differences were found between the IIC (predictable vs. unpredictable) task strategies.

A literature review revealed an absence of previous studies of central and peripheral motor fatigue in persons with MS based on IIC strategies using constant, predictable, and unpredictable motor tasks. We hypothesized that the nervous systems of these patients would be more fatigable than those of healthy men during the performance of exercises; thus, patients with MS would tend to experience: (a) higher central motor fatigue by performing IIC tasks with submaximal intensity, which would lead to an increase in motor variability and a higher number of errors; and (b) a higher central motor fatigue and motor variability by performing IIC tasks using an unpredictable strategy vs. performing IIC tasks using constant and predictable strategies.

## 2. Materials and Methods

### 2.1. Subjects

Two groups of subjects were recruited: 15 healthy young men (age range, 19–27 years) and nine male patients with MS (age range, 21–43 years; disease duration, 7.1 ± 3.0 years). All subjects had normal and/or corrected vision. Written informed consent was obtained from all participants after receiving clarification of all details of the investigational actions and the related inconveniences. All procedures were approved by the Human Research Ethics Committee and were conducted according to the guidelines of the Declaration of Helsinki. The Kaunas Regional Ethics Committee approved this study (No. BE-2-35). The criteria for inclusion in the MS group were a Kurtzke Expanded Disability Status Score (EDSS) < 4 [18] and a Fatigue Severity Scale (FSS) Score > 5 [19]. Fatigue was quantified using the MS-specific questionnaire (FSS), which contains nine items rated on a 7-point Likert scale. Disease in the MS group was of the relapsing–remitting type, according to the McDonald criteria [20].

All volunteers participated in recreational activities two or three times per week and were considered physically active, without taking part in any recognized physical exercise or sports program. All volunteers had a leading right leg, as assessed by self-reports of their favored kicking leg. The participants were nonsmokers and had no history of significant knee injury or surgery, pain during resisted knee extension, pain, or instability during functional activities, or fracture of the pelvis, femur, tibia, fibula, or patella within the previous two years. We excluded patients who had other neurological or psychiatric malfunctions, such as depression or anxiety, due to their recognizable connection with fatigue [21]. Individuals using prednisone or antispasmodic drugs were also excluded.

### 2.2. Experimental Design

A week prior the experimental trial, all subjects participated in an acquaintance session. Prior to the arrival at the laboratory, anthropometric variables were measured, and the experimental procedures for neuromuscular examination were demonstrated. All volunteers learned to achieve and maintain a maximal effort of the knee extensor for 3–4 s with a 250 ms test train (TT) of stimulation at 100 Hz (TT-100Hz) superimposed on a maximal voluntary contraction (MVC) to assess their tolerance to electrical stimulation (section “Isometric torque and electrical stimulation”). The volunteers also learned to maintain a force target at different torque intensities. They were tutored to refrain from consuming any food for a minimum of 12 h, to refrain from consuming alcohol and caffeine and engaging in heavy workout for a minimum of 24 h, and to sleep for a minimum of 8 h prior to each investigational session. To standardize the state of hydration and the sense of thirst, the subjects could take still water as desired up until 60 min prior to the testing. Examinations were performed at a laboratory temperature of 21 °C and 50% relative humidity.

### 2.3. Experimental Protocol

The protocol involved three differently organized exercise task sessions, each of which was carried out randomly and separated by a rest period of a minimum of 7 days. The tasks were performed indoors starting at the same time of day (7 AM), to minimize body temperature. Prior to the arrival at the laboratory, the participants were asked to wear short-sleeved shirts, shorts, and socks, and to lie down in a horizontal position for 10 min. After the relaxation period and before the exercises began, intramuscular temperature (T_mu_) was measured.

Prior to the start of each trial, the subjects performed a 10 min warm-up on an electrically braked cycle ergometer (Ergo-Fit, Pirmasens, Germany) with a pedaling frequency that ranged between 60 and 70 rpm. The subjects were seated in a dynamometer chair, and stimulating electrodes were positioned over the quadriceps femoris muscle of their right leg. Subsequently, the force-generating capacity of the quadriceps muscle was evaluated by applying 1 s trains of electrical stimuli at 20 Hz and 100 Hz. Nearly 3 s were required to change the frequency of stimulation. After a 20 s break, two 5 s MVCs were assed with a 20 s rest period between them. At about 3 s of MVC, a 250 ms TT-100 Hz was superimposed on the voluntary contraction. These TT-100 Hz contractions were used to determine the voluntary activation of the knee extensors. MVC force was defined as the maximum force that could be sustained for 2–3 s. If the relative difference between these two MVCs was within 10%, no further trial was needed [22].

**Figure 1:**
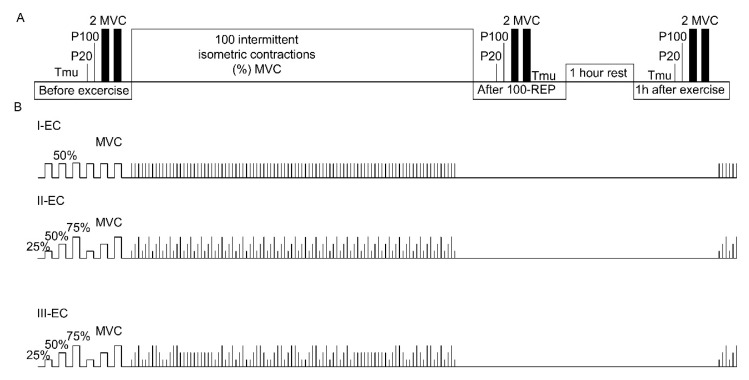
Experimental protocol. (**A**) Scheme of the experimental protocol; (**B**) Structural pattern illustration of the task for the three experimental conditions (ECs). Maximal voluntary contraction (MVC) torque, intramuscular temperature (T_mu_) and electrically induced quadriceps femoris muscle isometric torque caused by stimuli at 20 Hz (P20), 100 Hz (P100).

The higher of the two MVCs was used to compute the submaximal target torque. The percentage torque to be reached and sustained (“target torque”) was indicated by a horizontal line on the screen of the dynamometer. We set the visual refreshing rate frequency at 2 kHz on a standard monitor (Dell P2717H); the refresh rate was 60 Hz, the resolution was full high definition (1920 × 1080 pixels), the brightness/contrast ratio was 1:1, and a continuous line signal was provided. The participants then performed the IIC tasks under one of the three experimental conditions (ECs; constant, predictable, and unpredictable). All three IIC ECs included 100 isometric contractions, followed by a 1 h rest period and six IICs. The neuromuscular variables, i.e., electrical stimulus, MVC, central activation ratio (CAR), and T_mu_, were measured three times in each session: before exercise, after 100 contractions (repetitions or Reps), and 1 h after exercise (Figure 1A).

### 2.4. The Three IIC ECs

In each set of 100 Reps, knee extension was sustained for 5 s, and the muscle was then allowed to relax for 20 s. The torque output sum of 100 Reps under the three different IIC strategies was the same and corresponded to a mean intensity of 50% MVC. The main difference between the IIC tasks was the combination of muscle contraction torque intensity positions. Evidence indicates that the repeated performance of intermittent contractions during constant or predictable (i.e., stable) torque target sequence tasks elicits a learning effect. In the context of this monotonically adapted or learned motor task, unexpected (unpredictable) changes in the torque level in a selected task segment produce an unstable motor behavior, and greater cognitive–motor resources are required to perform the correct task-directed effort [23]. In consideration of this, the first EC (I-EC) comprised a constant torque target sequence at 50% of MVC; the second EC (II-EC) comprised an IIC task with a predictable torque target sequence at 25%, 50%, or 75% of MVC; and the third EC (III-EC) involved an unpredictable torque target sequence at 25%, 50%, or 75% of MVC. After each of the EC tasks, six other IICs were performed to provide an indication of muscle contraction steadiness after resting (Figure 1B).

Prior to and throughout each trial, the subjects were not introduced to the structure or sequence of the ECs and were blindfolded to block their view of the video display screen. The blindfold was then removed, and the participant had to perform the IIC to reach the upper torque line seen on the video display screen as quickly as possible and sustain the position for 5 s as smoothly and precisely as possible. Following the attempt, the covering was placed over their eyes and the participant rested for 20 s before the subsequent attempt. Throughout resting, the torque line was changed with reference to the EC protocol sequence, and the participant had no knowledge of where the torque line would appear on the next attempt.

### 2.5. Measurements

Throughout the initial visit, the height (cm) of subjects was measured, and their weight (kg), body mass index (kg/m^2^), fat-free mass (kg), and body fat (%) were estimated by taking their nude body mass using a body composition analyzer (TBF-300; Tanita, UK Ltd., West Drayton, UK) (Table 1).

### 2.6. Isometric Torque and Electrical Stimulation

The isometric torque of knee extensor muscles was measured using an isokinetic dynamometer (System 3; Biodex Medical Systems, Shirley, NY, USA), and data were acquired using dedicated software (Version 2.15; Biodex Medical Systems, Shirley, NY, USA). The subjects sat upright in the dynamometer chair with their knees positioned at a 60° flexion (0° full extension). Their shank, trunk, and shoulder were stabilized by belts. VF of torque from the dynamometer was provided on a video display screen located in front of the participants during the trial. The screen was positioned at eye level about 1 m in front of the participants. Electrical stimulation was applied directly to the muscles using two carbonized rubber electrodes covered with a thin layer of electrode gel (ECG–EEG Gel; Medigel, Modi’in, Israel). One of the electrodes (6 cm × 11 cm) was placed transversally across the width of the proximal portion of the quadriceps femoris. Another electrode (6 cm × 20 cm) covered the distal portion of the muscle above the patella. Stimulation was applied using a standard electrical stimulator (MG 440; Medicor, Budapest, Hungary) and was delivered in 0.5 ms square-wave pulses. Peak torques induced by 1 s of electrical stimulation at 20 Hz (P20; representing the steep section of the force–frequency relationship curve) and 100 Hz (P100; which is close to the maximal force) were measured with a 3 s rest interval between electrical stimulations [22,24]. The change in the P20 to P100 ratio (P20/P100) was used as a proxy of low-frequency fatigue [25]. The tolerance to electrical stimulation was evaluated during the introduction session, and only participants who exhibited good compliance with the procedure were employed in the study.

The intensity of the electrical stimulation was selected individually by applying single stimuli to the quadriceps muscle. The voltage was increased gradually, in 3 V increments. The voltage at which the participant first felt pain was defined as the pain threshold, and the voltage that could no longer be tolerated by the participant was defined as the pain tolerance [17]. During this procedure, the voltage was increased until no increment in single twitch torque could be detected with an additional 10% increase in the voltage.

After the tolerance to the electrical stimulation was evaluated at rest, two to three brief MVCs were required from subjects without superimposed TT-100Hz; subsequently, the same two to three brief MVCs, with a supplementary test trial (TT-100Hz) superimposed on an MVC, were terminated. Thus, if the relative difference between MVCs without superimposed TT-100Hz and MVCs with superimposed TT-100Hz was within 10%, the tolerance to TT-100Hz during brief MVCs was indicated as acceptable [22].

### 2.7. Central Activation Ratio (CAR) Measurements

The CAR is the ratio of the maximal voluntary torque to the peak torque generated with a supplementary TT-100 Hz superimposed on an MVC. To evaluate the voluntary activation of knee extensors, a 250 ms trial of stimuli at 100 Hz (TT-100 Hz) was superimposed on the voluntary contraction at about 3 s of MVC. The CAR was computed as defined elsewhere [2,22,26] using the following equation:

CAR = MVC/(MVC + TT − 100 Hz) × 100%(1)

where a CAR of 100% indicates complete activation of the exercising muscle and a CAR < 100% indicates central activation failure or inhibition.

### 2.8. Muscle Temperature Measurements

T_mu_ was evaluated using a needle microprobe (MKA; Ellab, Hvidovre, Denmark) inserted 3 cm under the skin and covering the vastus lateralis muscle of the right leg [27]. Skin preparation prior to each T_mu_ measurement involved skin shaving and disinfection with a cotton–wool tampon soaked in isopropyl alcohol before and after insertion. Insertion was made without any anesthesia. After the first measurement, the insertion area was marked with a circle with a diameter of 0.5 cm.

### 2.9. Rating of Perceived Exertion (RPE)

The subjects were requested to rate their perceived exertion on the Borg scale, with values ranging from 6 (no exertion at all) to 20 (maximal exertion). The RPE was measured with VF at 50% of the required torque at the time points (before exercise, after 100 Reps, and 1 h after exercise).

### 2.10. Accuracy and Motor Variability of IIC Assessment

The accuracy of the 5 s target IIC torque at 50% of MVC was computed as the constant error and absolute error; however, the coefficient of variation was computed to estimate the intraindividual motor variability of 5 s IICs. Attention was paid to algebraic symbols (±). The absolute error specifies the absolute deviation from the required target force (50% of the isometric force required).

Constant error = ∑(*x_i_* − *T*)/*n* (2)

where *x_i_* is the IIC performed (N·m); *T* is the target quantity, i.e., the IIC required; *n* is the number of trials; and Σ indicates the mean that was calculated considering the algebraic symbols (±).

Absolute error = ∑|*x_i_* − *T*|/*n* (3)

where *x_i_* is the IIC performed (N·m); *T* is the target quantity, i.e., the IIC required; *n* is the number of trials; and vertical brackets Σ | | indicate the mean that was calculated without considering the algebraic symbols (±).

(4) $$\mathrm{Coefficient}\;\mathrm{of}\;\mathrm{variantion}\;(\mathrm{normalized}\;\mathrm{variable}\;\mathrm{error})=(\mathrm{SD}/\bar{x})\times 100$$

where SD is the mean standard deviation of IICs performed; $\bar{x}$ and is the mean IIC (N·m).

### 2.11. Statistical Analyses

The normality of the data distribution was evaluated using the Kolmogorov–Smirnov test; all data were found to be normally distributed, except the Borg scale. Single-time-point comparisons between groups were analyzed using independent sample *t-*tests for the baseline values (physical characteristics).

Repeated-measures analysis of variance (ANOVA) was used to study the effects of the three ECs (I-EC vs. II-EC vs. III-EC) as a within-subject factor for T_mu_, the accuracy and variability of IIC, CAR, MVC, and the electrically induced torque before exercise.

A mixed ANOVA was performed with one between-subjects factor (groups: healthy men vs. MS men) and multiple within-subject factors (I-EC vs. II-EC vs. III-EC) and time (before exercise vs. 100 Reps vs. 1 h after exercise) for electrically induced torque, T_mu_, MVC and CAR, the coefficient of variation, and constant and absolute errors (i.e., the accuracy and variability of IIC).

If significant effects were found, Sidak’s post-hoc adjustment was used for multiple comparisons within each repeated-measure ANOVA. Calculations of statistical power (SP, as a percentage) were performed for all indicators based on an alpha level of 0.05, sample size (*n* = 11), standard deviations, and changes in the average level of the data. The SP for a significant effect was >80%. The partial eta squared (*η_p_*^2^) was estimated as a measure of the EC effect size. The nonparametric Wilcoxon signed rank test for two related samples was used to compare changes in subjective ratings of effort sensation. Descriptive data are presented as the mean ± standard deviation. The significance of all tests was set at *p* < 0.05. Data were analyzed using SPSS version 21.0 (IBM Corp., Armonk, NY, USA).

## 3. Results

### 3.1. Participants’ Characteristics

Table 1 describes the reference characteristics of the participants. Healthy men and men with MS had similar height, mass, BMI, fat-free mass, body fat %, and age, with significant differences between groups (*p* < 0.05) (Table 1).

### 3.2. Baseline Neuromuscular and Intramuscular Temperature (T_mu_) Characteristics

Table 2 presents the baseline data of MVC torque, CAR, and T_mu_. Generally, men with MS were weaker than healthy men. Table 2 shows that, for the initial values obtained in all EC tasks, men with MS had lower MVC torque, CAR, and T_mu_ compared with healthy men (*p* < 0.001, *η_p_*^2^ = 0.54, SP > 99%, *p* < 0.05, *η_p_*^2^ = 0.22, SP > 80%, and *p* < 0.05, *η_p_*^2^ = 0.33, SP > 85%, respectively). No differences were observed between the initial values of men with MS and healthy men in before-exercise MVC torque, CAR, or T_mu_ among all EC tasks (*p* > 0.05) in (Table 2).

### 3.3. Maximal Voluntary Contraction (MVC ) Torque, Intramuscular Temperature(T_mu_), Central Activation Ratio (CAR) and Rating of Perceived Exertion (RPE)

**Figure 2:**
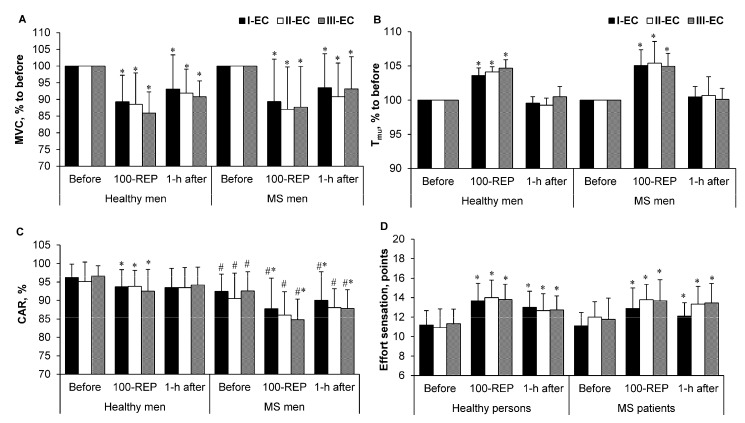
(**A**) Maximal voluntary contraction (MVC) torque and (**B**) intramuscular temperature (T_mu_) after 100 repetitions (100-REP) and at 1 h after exercise in the three experimental conditions (ECs). Values of MVC torque and T_mu_ < 100% were taken from the initial measurements in these experiments. (**C**) Central activation ratio (CAR) and (**D**) effort sensation values before, after 100 repetitions (100-REP), and at 1 h after exercise in the three ECs. Values are expressed as means ± standard deviation. * *p* < 0.05 compared with “before exercise”; ^#^ *p* < 0.05 when comparing healthy men and men with multiple sclerosis (MS).

In all EC tasks, MVC torque diminished after 100 Reps and remained unrecovered 1 h after exercise compared with the values recorded before exercise (*p* < 0.001, *η_p_*^2^ = 0.8, SP > 95%; and *p* < 0.05, *η_p_*^2^ = 0.6, SP > 85%, respectively) for both healthy controls and men with MS (Figure 2A).

Figure 2B shows that the T_mu_ for both healthy controls and men with MS increased after 100 Reps (*p* < 0.001, *η_p_*^2^ = 0.98, SP > 99%; and *p* < 0.001, *η_p_*^2^ = 0.85, SP > 99%, respectively) and returned to before-exercise values after 1 h of recovery (*p >* 0.05) in all EC tasks.

In all EC tasks, the CAR of healthy men decreased after 100 Reps (*p* < 0.05, *η_p_*^2^ = 0.75, SP > 80%) and returned to before-exercise values after 1 h of recovery. The CAR of men with MS decreased after 100 Reps and remained unrecovered to pre-exercise values within 1 h after the exercise in the I-EC and III-EC trials (*p* < 0.05, *η_p_*^2^ = 0.4, SP > 80%; *p* < 0.05, *η_p_*^2^ = 0.5, SP > 85%, respectively) (Figure 2C). Conversely, the II-EC trial resulted in no differences in the CAR values of the 100 Reps and 1 h after exercise conditions compared with the values recorded before exercise. In all EC tasks, the CAR values of healthy and MS subjects were insignificantly different between groups (*p* < 0.05, *η_p_*^2^ = 0.33, SP > 85%) (Figure 2C).

In all EC tasks, the subjective sensation of effort for both healthy and MS subjects increased after 100 Reps and remained constant at 1 h after exercise (*p* < 0.001, *η_p_*^2^ = 0.9, SP > 99%; *p* < 0.05, *η_p_*^2^ = 0.6, SP > 95%, respectively) (Figure 2D).

However, a comparison of MVC torque, T_mu_, and the subjective sensation of effort in all EC tasks resulted in nonsignificant differences between the two groups. Figure 2 shows an absence of significant differences in MVC, T_mu_, CAR, and the subjective sensation of effort among the EC tasks (*p* > 0.05).

### 3.4. Electrically Induced Muscle Torque

**Figure 3:**
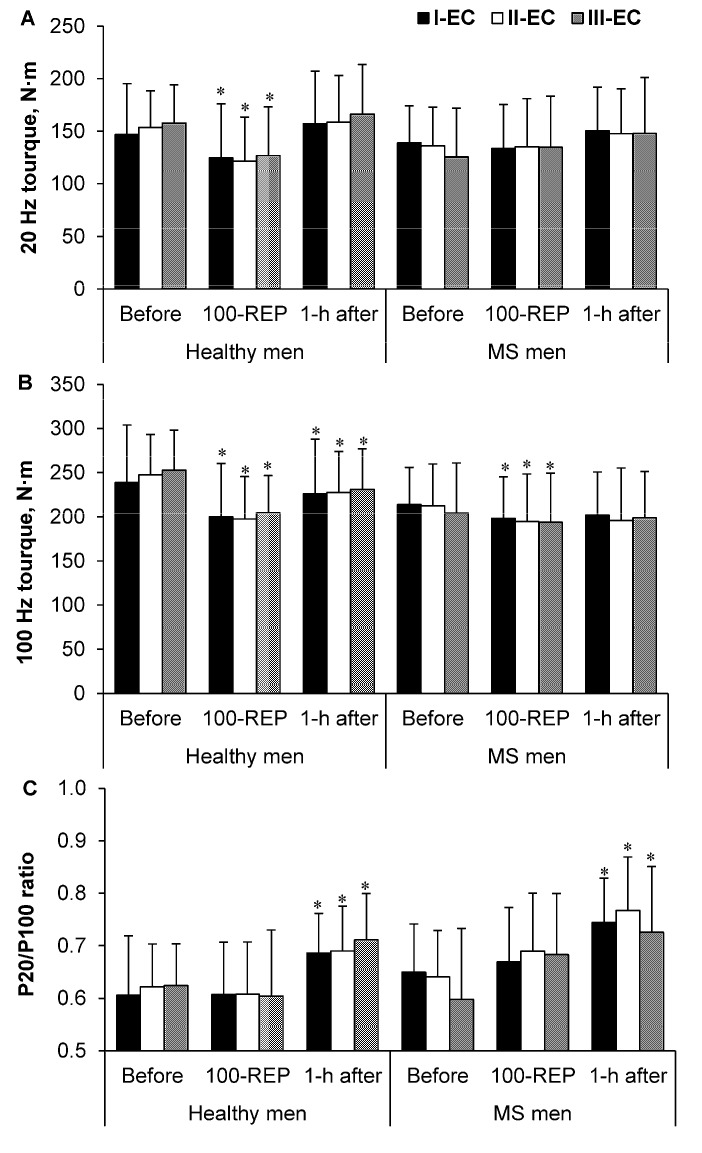
Electrically induced quadriceps femoris muscle isometric torque caused by stimuli at (**A**) 20 Hz; (**B**) 100 Hz; (**C**) the ratio of P20/P100 torques before exercise, 100 repetitions (100-REP), and at 1 h after exercise in the three experimental conditions (ECs). Values are expressed as means ± standard deviation. * *p* < 0.05 compared with “before exercise”.

For all EC tasks, the electrically induced (P20) torque of healthy men decreased significantly after 100 Reps (*p* < 0.001, *η_p_*^2^ = 0.62, SP > 99%) and had recovered to before-exercise values at 1 h after exercise (*p* > 0.05) (Figure 3A). In all EC tasks, in the MS group, a comparison of the (P20) torque values of 100 Reps and at 1 h after exercise with the initial ones recorded before exercise revealed an absence of differences (Figure 3A).

In the group of healthy men (P100), the torque decreased significantly after 100 Reps, without regression to the before-exercise level at 1 h after exercise, for all EC tasks (*p* < 0.001, *η_p_*^2^ = 0.68, SP > 99%) (Figure 3B). In the MS group (P100), the torque decreased significantly after 100 Reps (*p* < 0.05 *η_p_*^2^ = 0.47 SP > 85%), with regression to the before-exercise level within 1 h after the exercise (*p* > 0.05), for all EC tasks (Figure 3B).

In both groups, the P20/P100 ratio increased at 1 h after exercise (*p* < 0.001, *η_p_*^2^ = 0.6 SP > 99%; *p* < 0.05, *η_p_*^2^ = 0.6 SP > 90%, respectively) for all EC tasks (Figure 3C).

However, a comparison of (P20) with (P100) and the ratio of P20/P100 torques in all EC tasks resulted in nonsignificant differences between the two groups (Figure 3A–C). Figure 3 shows the absence of significant differences in electrically induced quadriceps femoris muscle isometric torque at (P20), (P100), and the ratio of P20/P100 torques among all EC tasks (*p* > 0.05).

### 3.5. Accuracy and Motor Variability of IIC Tasks

Table 3 presents the data on the coefficient of variation, constant error, and absolute error values. The variation coefficient, constant, and absolute errors of 50% isometric MVC did not differ significantly between the groups of healthy and MS subjects (*p* > 0.05). We found no effect of EC tasks on the variation coefficient, constant, or absolute errors (Table 3).

## 4. Discussion

The main findings of our study are as follows:

(1) The decrease in MVC force was the same for both groups; however, the decrease observed in MS subjects occurred via central neural mechanisms, whereas in healthy men, it occurred through peripheral components;

(2) The CAR was lower for individuals with MS than for healthy men, although the effort sensation increased evenly and with no reference to IIC strategies;

(3) The central and peripheral motor fatigue of MS and healthy subjects had no reference to IIC strategies;

(4) No difference was observed in motor variability or movement accuracy in men with MS or healthy men in all three ECs considered. Neither the first nor second of our hypotheses were confirmed by the findings of our study. The first hypothesis was that men with MS would perform IIC with higher motor variability and a higher number of errors compared with healthy men. However, we found that, in all three considered ECs, the CAR of men with MS was lower than that of healthy men (Figure 2C). Moreover, it was assumed that a decrease in the CAR would lead to an increase in the coefficient of variation, and absolute and constant errors; however, the study yielded the opposite results, i.e., with any decrease of the CAR, no difference was seen in the coefficient of variation or absolute and constant errors in either men with MS or healthy men (Table 3). The absence of variation in these parameters could be indicative of the interaction between learning and fatigue. In the process of motor learning, the variability of movements decreases, and accuracy increases [28]; the increase in fatigue leads to the contrary tendency [2].

The second hypothesis assumed that throughout the performance of IIC tasks using the unpredictable strategy, the men with MS would experience higher central fatigue (a higher decrease of the CAR), which would increase the number of errors and motor variability. However, our results revealed that central motor fatigue, the number of errors, and variability were not correlated with IIC task strategy (Figure 2C). The learning of movements during the performance of IIC tasks could counterbalance the appearance of central fatigue [28]. Unfortunately, the study failed to distinguish the effects of each of those two processes.

Our findings reflect the results of previous research, indicating that during the performance of physical exercise, patients with MS experience higher central but lower peripheral fatigue [3,8,9,10]. Moreover, peripheral fatigue, i.e., decreases in electrically induced force, also depends on two processes: interactions of muscle potentiation, and fatigue [4,29]. Previous studies have shown that the performance of 100 repetitive maximal voluntary quadriceps muscle contractions leads to assertion of post-tetanic potentiation, low-frequency fatigue, and post-contractile depression [29].

Electrically induced isometric force production at 20 Hz (P20) did not exceed the value of 100 Hz (P100) force production (Figure 3A,B); thus, no evidence of low-frequency fatigue was seen. Increased muscle temperature evokes a decrease in involuntary muscle force [27]. In addition, during the time of recovery, an increase in the P20/P100 factor was observed in both groups, indicating a muscle potentiation mechanism before evidential fatigue [4]. This muscle potentiation mechanism may conceal an increase in fatigue-based muscle force sensation, i.e., a higher potentiation leads to a lower force sensation [30]. With respect to the predominance of chronic fatigue, men with MS [31] require potentiation of the motor system. Based on previous observations, the evidence of muscle potentiation during the performance of IIC tasks can be applicable as a form of rehabilitation, whereas peripheral fatigue does not differ between healthy and multiple sclerosis working-age adults, significantly affecting their daily efficiency.

## 5. Conclusions

The results of the present study revealed that men with MS exhibited higher central motor fatigue than did healthy men, although this had no effect on the variability, accuracy, or force sensation of the performed movements. This might be the result of muscle potentiation, motor learning, or motor fatigue. Consequently, because of motor fatigue potentiation on their muscles, men with MS could accomplish submaximal IIC, resulting in better task performance.

## Figures and Tables

**Table 1:** Physical characteristics of the participants.

	Healthy Men (*n* = 15)	MS Men (*n* = 9)
Age, yr.	22.47 ± 3.74	31.11 ± 8.51 ^#^
Height, cm	182.53 ± 6.77	178.67 ± 5.79
Mass, kg	78.51 ± 8.09	73.13 ± 6.72
Body mass index, kg/m^2^	23.58 ± 2.29	22.90 ± 1.53
Fat free mass, kg	12.67 ± 4.34	12.42 ± 3.99
Body fat, %	15.86 ± 4.15	16.77 ± 4.46
EDSS, points		3.1 ± 1.3

Expanded Disability Status Score (EDSS). Values are means ± standard deviation. ^#^ *p* < 0.05 when comparing healthy men and men with multiple sclerosis.

**Table 2:** Reference characteristics of maximal voluntary contraction (MVC) torque, central activation ratio (CAR), and intramuscular temperature (T_mu_) in healthy men and men with multiple sclerosis (MS) before exercise in 3 experimental conditions (ECs).

		MVC (N·m)	CAR (%)	T_mu_ (°C)
**I-EC**	Healthy men	316.79 ± 44.42	96.22 ± 3.65	36.9 ± 0.5
	MS men	234.51 ± 39.83 ^#^	92.44 ± 4.72 ^#^	36.2 ± 0.5 ^#^
**II-EC**	Healthy men	310.83 ± 40.16	95.15 ± 5.25	36.9 ± 0.4
	MS men	228.84 ± 41.37 ^#^	90.55 ± 6.87 ^#^	36.0 ± 1.0 ^#^
**III-EC**	Healthy men	320.28 ± 36.40	96.57 ± 2.83	36.7 ± 0.6
	MS men	231.57 ± 47.88 ^#^	92.55 ± 5.22 ^#^	36.3 ± 0.6 ^#^

Values are means ± standard deviation. ^#^ *p* < 0.05 when comparing healthy men and men with MS.

**Table 3:** Coefficient of variation (CV), constant error (CE), and absolute error (AE) values before exercise, after 100 repetitions (100-REP), and 1 h after exercise in the three ECs.

		CV (%)		
		Before Exercise	After 100-REP	1-h after Exercise
**I-EC**	Healthy men	4.1 ± 1.7	3.5 ± 1.5	3.3 ± 1.5
	MS men	6.1 ± 3.5	3.0 ± 1.9	2.9 ± 2.5
**II-EC**	Healthy men	5.6 ± 3.7	5.0 ± 2.1	3.9 ± 1.6
	MS men	5.1 ± 3.6	2.9 ± 1.4	4.8 ± 4.8
**III-EC**	Healthy men	6.8 ± 8.3	3.4 ± 1.1	3.4 ± 2.0
	MS men	3.9 ± 2.6	3.3 ± 1.5	4.7 ± 3.6
		**CE (N·m)**		
**I-EC**	Healthy men	−4.4 ± 4.9	−2.1 ± 6.4	−2.6 ± 5.9
	MS men	−5.21 ± 4.01	−5.07 ± 5.17	−6.39 ± 5.86
**II-EC**	Healthy men	−8.8 ± 6.7	−3.2 ± 5.8	−3.9 ± 4.9
	MS men	−7.31 ± 5.64	−5.14 ± 6.92	−7.44 ± 5.68
**III-EC**	Healthy men	−7.4 ± 8.9	−3.5 ± 5.2	−6.6 ± 9.9
	MS men	−6.87 ± 5.09	−4.23 ± 4.11	−7.63 ± 4.27
		**AE (N·m)**		
**I-EC**	Healthy men	7.1 ± 2.6	6.4 ± 1.9	6.4 ± 2.9
	MS men	7.70 ± 2.37	6.89 ± 2.10	7.89 ± 3.74
**II-EC**	Healthy men	10.2 ± 4.6	7.0 ± 2.1	6.5 ± 2.3
	MS men	7.61 ± 5.42	7.49 ± 4.42	7.69 ± 5.39
**III-EC**	Healthy men	9.3 ± 8.1	6.1 ± 3.6	7.4 ± 9.6
	MS men	7.48 ± 4.00	5.88 ± 1.94	8.14 ± 4.31

Data are shown as means ± standard deviation.
